# Division of labor in work shifts by leaf-cutting ants

**DOI:** 10.1038/s41598-021-88005-0

**Published:** 2021-04-22

**Authors:** Pedro B. Constantino, Veronica S. Valentinuzzi, André F. Helene

**Affiliations:** 1 Department of Physiology, Instituto de Biociências da Universidade de São Paulo (IB-USP), São Paulo, SP 05508-090 Brazil; 2 Centro Regional de Investigaciones Científicas y Transferencia Tecnológica de La Rioja (CRILAR), UNLAR, SEGEMAR, UNCa, CONICET, Anillaco, La Rioja Argentina

**Keywords:** Behavioural ecology, Ecophysiology, Circadian rhythms and sleep, Social behaviour

## Abstract

Foraging rhythms in eusocial insects are determined by the colony´s overall pattern. However, in leaf-cutting ant workers, individual rhythms are not fully synchronized with the colonies’ rhythm. The colony as a whole is nocturnal, since most worker activity takes place at night; however some workers forage during the day. Previous studies in individualized ants suggest nocturnal and diurnal workers coexistence. Here observations within the colony, in leaf-cutting ants, showed that workers have differential foraging time preference, which interestingly is associated to body size and differential leaf transportation engagement. Nocturnal ants are smaller and less engaged in leaf transportation whereas diurnal ants are bigger and more engaged in leaf carriage. Mechanisms underlying division of labor in work shifts in ants are still unknown but much can be extrapolated from honeybees; another social system bearing a similar pattern. A collective organization like this favors constant exploitation of food sources while preserving natural individual rhythm patterns, which arise from individual differences, and thermal tolerance, given by the size polymorphism presented by this species.

## Introduction

Eusocial insect behavior is commonly analyzed in two distinct but not independent biological levels; these are (1) individual and (2) collective^1–6^. The first concerns behavior of individuals as autonomous entities, owners of a nervous system, capable of information integration and responsiveness to environmental stimuli^7–10^. The second concerns the colony as a unit capable of complex collective behaviors that may or may not directly reflect the behaviors of each individual^11–14^.

Self-organization models settle as a powerful tool to explain how collective behaviors emerge from countless interactions between individuals within a colony^15,16^. Individual behaviors are treated as a simple response to the environment in these models^17^. However, such assumption does not allow a deeper evaluation of the physiological and behavioral aspects of the individual.

Foraging is an example of a collective behavior displayed by colonies that involve individual behavioral components^2,6,18,19^. Colonies of social wasps, bees and ants have a preferential foraging phase that describes species as nocturnal or diurnal^20–26^. It is expected that the relationship between light–dark cycles and foraging rhythms to be given by the physiological system of endogenous oscillator^27^. These oscillators are typically based in self-regulated clock genes feedback loops^28,29^. Since genes are involved, the foraging rhythm would be expected to be inheritable, individual and common to all individuals, as workers are siblings and have a higher degree of kinship due to haplodiploid sexual determination^30,31^. However, this is not the case in leaf-cutting ants.

Leaf-cutting ants build long foraging trails between nests and food sources^32–34^. Previous studies described the foraging pattern of *Atta cephalotes* as nocturnal, since foraging trails had higher ant flows during the dark phase^35,36^. However a small, but not negligible number of ants were observed during the light phase. Either these ants are arrhythmic or the workers are able to divide labor in work shifts.

Chronobiological experiments showed evidence that *Camponotus compressus* ants have the endogenous mechanism required to divide labor in work shifts^37^. Despite the fact that these data was collected in isolated individuals, which can disrupt the natural behavior of a social organism^38^, it is important evidence of a complex mechanism that underlies individual behavior to collective synchronization.

In the present work another approach was used to identify if leaf-cutting ants are work shifters. To do so, groups of ants from laboratory kept colonies (12:12 LD cycle) were marked in four distinct 2-h shifts (two during the light phase and two during dark phase). The distribution of these marked ants was then observed for the following five consecutive days after marking. Other variables measured were total ant flow, to characterize the colony’s foraging rhythm, and total leaf transportation to measure foraging efficiency during the different phases. Since leaf-cutting ants have a high degree of polymorphism^39,40^, ant size was also quantified and the relationship between size, physiology and behavior was proposed to explain the results.

## Materials and methods

**Figure 1:**
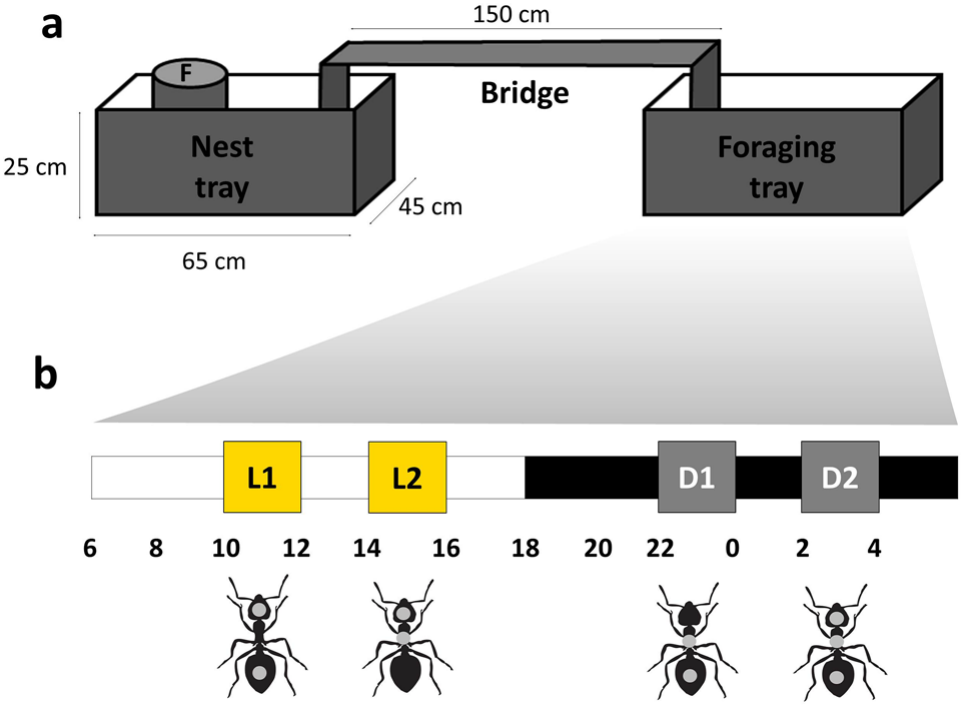
Experimental setup schematics. **(a)** Spatial disposition of colonies areas, highlighting nest tray, connective bridge and foraging tray. **(b)** Selected work shifts from 24 h period: L1 (10:00–12:00) and L2 (14:00–16:00) and during the dark phase D1 (22:00–00:00) and D2 (02:00–04:00). Note that work shifts are far from crepuscular phase and are disposed 2 h apart in same lighting condition and 12 h apart in opposite light condition.

Six *Atta sexdens* colonies kept in laboratory were used. Two weeks before the beginning of the experiment, each colony was moved to the test room for adaptation to a highly controlled environmental condition—temperature: 25  ± 1 ºC; relative humidity: 70 ± 2%; photoperiod: 12:12 LD (lights on at 06:00 h). The colonies were kept in plastic trays (H × L × W: 65 × 45 × 25 cm) and the fungus garden was raised in a plastic cylinder (D × L: 24 × 25 cm) inside the plastic tray. A 1.5 m wooden bridge connected the colony tray with a same size but empty tray, hereinafter referred as foraging tray. Fresh *Acalipha sp*. leaves were offered ad libitum as food source (Fig. 1). Experiments were 6 days long: one marking day and five observation days.

### Marking

Ant marking took place in what will be called day 0. Four, 2-h-long intervals (called “work shifts”) were selected within a 24 h cycle as follows—During the light phase: L1 (10:00–12:00) and L2 (14:00–16:00); and during the dark phase: D1 (22:00–00:00) and D2 (02:00–04:00). These shifts were chosen to avoid proximity to the dark–light and light–dark transitions. During each selected shift interval, loaded ants returning from the foraging tray were removed from the trail and manually marked with a SHARPIE oil-based ink^41^. Each group was marked with a unique pattern allowing identification in posterior observations.

### Observation

At the end of the foraging trail a LOGITECH c920 HD Pro Webcam connected to a computer running the surveillance software ISPY was placed (Fig. 1). The schedule function was used to record 2-h-long videos during the same work shifts when marking took place. Videos were recorded daily during the five consecutive days that followed marking. For night recording we used red LED lamps that only emit electromagnetic waves of > 650 nm, and less than 1 lx of intensity, which are invisible to ants, since they do not have photoreceptors to this wavelength^42^.

Videos were analyzed collecting the following data: (1) total number of marked ants from each group that crossed the bridge; (2) total number of leaves carried. These data allowed comparison of the number of marked ants in each shift within a day and between days. In each video, an (3) estimated value of the total flow was obtained by counting the total number of ants that crossed the bridge during 12 random minutes within the 120 min video interval.

A correlation between ant flow and leaf transportation was made using data from total number of leaves carried and the total estimate ant flow for each light or dark condition. This correlation, here named foraging efficiency, allows analysis of the colony’s retrieving food effort and how it varies during the different phases.

### Ant measurements

During the observations it appeared that ants marked on light phase and night phase were from different sizes. Since leaf-cutting ants presents a high degree of polymorphism it seemed reasonable to assume that this difference could have a biological significance. Thus, ants were digitally measured on replica 5 and 6 since a metric scale was placed in the webcam field view, to test the hypothesis of morphological difference among day ants and night ants. This metric scale was used to standardize pixel length obtained from IMAGEJ measure function taken from whole body of ants passing through. Body length from 705 ants distributed on the four shifts during the five testing days was measured using IMAGEJ software. Those 705 ants were selected from 400 frames of videos from 2 out of 6 replicas.

### Statistical analyses

Collected data were from observed ants in different shifts and days. Since data were not normally distributed, to compare the number of ants observed in different shifts and days, a set of generalized linear mixed model (GLMM) were fitted using a Poisson distribution. Number of marked observed ants was the response variable and as fixed effects it was used ‘work shift’ (L1, L2, D1 and D2) and ‘observation day’ (1, 2, 3, 4 and 5); experimental colony was used as random factor. The model that accounted for interactions between fixed factors was used, since there was this kind of interactions (see results below). Following with the model analysis a post-hoc test was made to determine overall patterns within fixed factors. To analyze overall ant flow regardless of marked ants a Kruskall-Wallis (KW) test was performed with a Dunn post-hoc test for multiple comparisons between groups. Linear regression analyses were performed to compare the correlation between total estimated ant flow and total leaf transportation during the light or dark phase. Ant size data were normally distributed, so analysis of variance (ANOVA) was performed followed by a Tukey post-hoc analyses for multiple comparisons. All the graphs and analyses were performed in R version 3.5.1. Anova, Tukey’s post-hoc test, Kruskall–Wallis test and linear regression analyses were available in basic stats package. Dunn’s post-hoc test was available in ‘FSA’ package. GLMM analyses were performed using the ‘glmer’ function in ‘lme4’ package and ‘emmeans’ function in ‘emmeans’ package for post-hoc tests. Graphs were made using ‘ggplot2’ package.

## Results

### Collective foraging characterization

**Figure 2:**
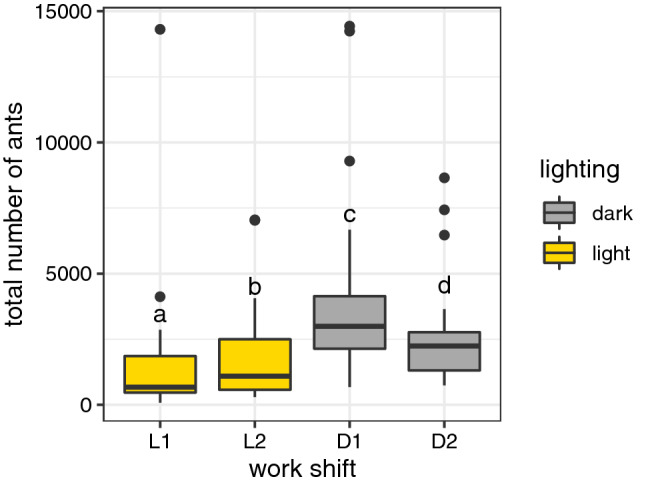
Total ant flow, regardless of marked ants, observed in each work shift for the 5 observation days. Boxplots represents the distribution of marked ants observed for the 5 day period and for the 6 colonies analyzed. Note that ant flow was bigger during night, especially for work shift D1, than day (ant flow KW: χ^2^ = 33.324, df = 3, p < 0.001). Letters above boxplots indicate statistically different distributions in a Dunn’s post-hoc test of a Kruskal–Wallis test at p < 0.05.

The colony as a whole had a preferential phase with a strong nocturnal foraging activity: mean number of ant flow was 1659 individuals during the light phase (work shifts L1 and L2) and 3271 during the dark phase (work shifts D1 and D2). It is also possible to observe that total ant flow varied among the selected work shift intervals. Colony activity was bigger during work shift D1 (mean number of ants: 3952) and smaller during work shift L1 (mean number of ants: 1509) (ant flow KW: χ^2^ = 33.324, df = 3, p < 0.001) (Fig. 2).

### Number of marked ants

A total number of 678 ants were marked throughout 6 replicas. The distribution of marked ants by replica and work shift can be found at Table 1. There were an uneven number of ants marked in each replica. This is mostly due to foraging activity differences in different work shifts and also in different engagement in foraging activity for different lighting conditions (see “Discussion” below for a deeper evaluation).

**Table 1:** Number of marked ants in each work shift of each replica.

	L1	L2	D1	D2
Replica 1	30	46	48	19
Replica 2	21	17	14	9
Replica 3	49	29	61	29
Replica 4	23	31	25	23
Replica 5	31	12	15	15
Replica 6	35	39	28	29
Mean	31.5	29	31.8	20.7
SD	10.03	12.85	18.84	7.94

### Temporal division of foraging behavior in work shifts

**Figure 3:**
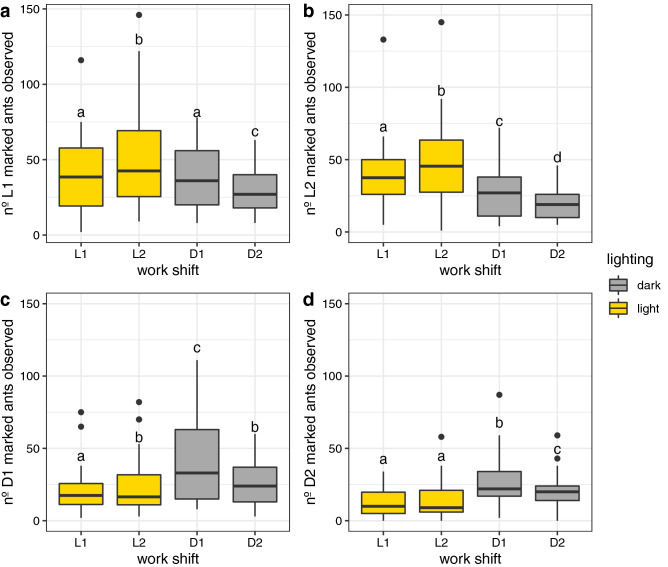
Distribution of marked ants in each work shift for the 5 observation days. Boxplots represents the distribution of marked ants observed for the 5 day period and for the 6 colonies analyzed. **(a)** Ants marked on L1 tended to forage at L2 work shift; **(b)** ants marked on L2 tended to forage at L2 work shift; **(c)** ants marked on D1 tended to forage at D1 work shift; **(d)** ants marked on D2 tended to forage at D1 work shift. Letters above boxplots indicate statistically different distributions using Tukey method for comparing a family of 4 estimates.

In Fig. 3a–d, it is possible to observe the distribution of marked ants in each selected work shift interval. The results show that both work shift and observation day had an effect on reappearance of marked ants (see below). Ants that were marked foraging at any of the four intervals during day 0 tended to forage during the same interval at the following five days. It is worth pointing out that lighting condition, rather than the work shift, was more important to explain reappearance.

Ants marked on L1 tended to forage more often in the L2 phase (GLMM: z = 22.68, p < 0.001), as does the ants marked on L2 (GLMM: z = 14.11, p < 0.001). Post-hoc analysis showed that both of these groups foraged less frequently at phase D2. On the other hand ants marked on D1 tended to forage more often in the D1 phase (GLMM: z = 11.69, p < 0.001), as does the ants marked on D2 (GLMM: z = 10.37, p < 0.001). Post-hoc analysis showed that both of these groups foraged less frequently at phase L1. There was a preferential work shift for foraging and another that was rejected in antiphase, that is, 12 h apart from each other. While during the intermediate work shifts, an intermediate foraging level was observed, as post-hoc analysis within fixed factor ‘work shift’ (letters above boxplots in Fig. 3).

GLMM analyses detected interactions between fixed factors ‘work shift’ and ‘observation day’. (Ants L1: work shift × observation day, z = − 6.085, p > 0.001; Ants L2: work shift x observation day, z = − 2.384, p = 0.017; Ants D1: work shift × observation day, z = 2.613, p = 0.008; Ants D2: work shift × observation day, z = 2.459, p = 0.013). Posterior analyses showed that this interaction is due to an overall reduction in reappearance of marked ants in later observation days regardless of the work shift. Thus, the effect of work shift is still a strong predictor of reappearance of marked ants.

### Foraging efficiency

**Figure 4:**
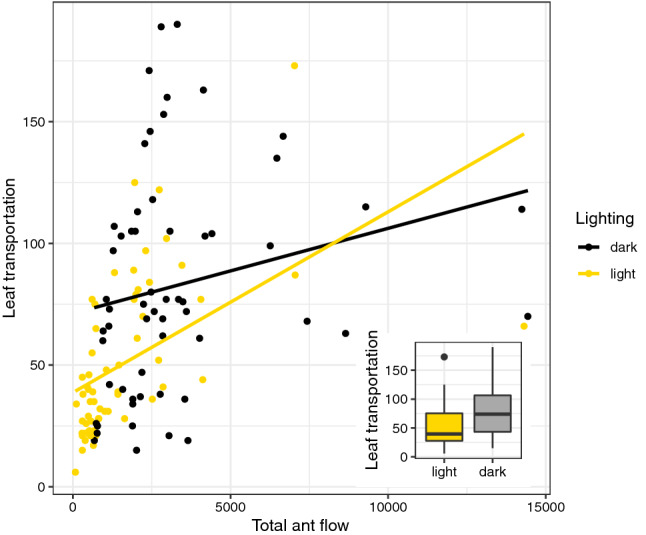
Leaf transportation as a function of total ant flow for the two lighting conditions. Note that foraging efficiency, given by the slope of the curves, varies between day and night (lm: F_(1, 116)_ = 18.926, p < 0.001). Foraging during day, although less frequent, is more efficient. Inserted boxplot indicates the amount of leaf transported during day and night.

A mean of 4.4% of returning ants were carrying a leaf fragment. Although it is a small proportion, it is consistent with previous observations of foraging efficiency in leaf-cutting ants. However, foraging efficiency, defined as proportional number of ants engaged in carrying (i.e., number of loaded returning ants as a function of total flow), changes with time of day. During daytime this proportion (5.4%) was consistently bigger than nighttime (3.4%) (Fig. 4) (lm: F_(1, 116)_ = 18.926, p < 0.001) meaning that there was a higher proportional engagement on food collection during the day relative to the night.

### Ant size

**Figure 5:**
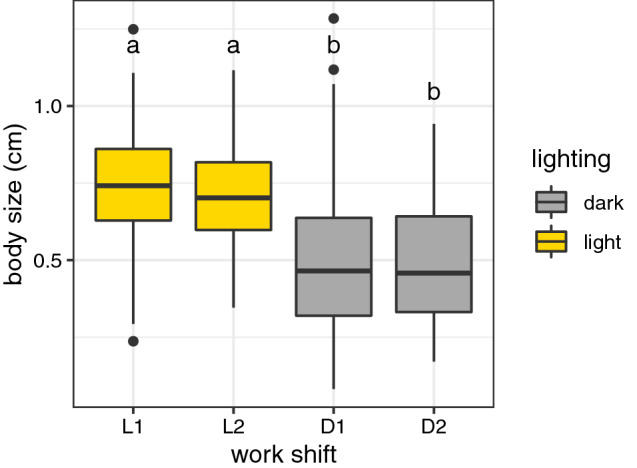
Boxplots showing body size (cm) distribution of ants measured in each selected work shift (ANOVA: F_(3, 701)_ = 88.04, p < 0.001). Letters above boxplots indicate statistically different distributions in a Tukey’s post-hoc test of an ANOVA.

During the four work shifts of the five observation days, 705 unmarked ants were measured. Day ants (work shifts L1 and L2) were consistently bigger in size than night ants (work shifts D1 and D2) (ANOVA: F_(3, 701)_ = 88.04, p < 0.001). As Fig. 5 shows there is no difference in size between work shifts in the same lighting condition. In other words, what seems to influence ant size is phase rather than the work shift.

## Discussion

Results clearly show two different colony activity states: (i) A high-flow nocturnal activity of smaller individuals but with a proportionally lower number of ants engaged in carrying leafs; and (ii) a diurnal low-flow activity of bigger ants but with a proportionally higher number of ants engaged in carrying. These results are in agreement with and expand data presented in previous work that show that *Atta sp.* colonies have a preferential nocturnal foraging behavior with a small but considerable number of ants foraging during the light phase^35,36^.

The observation of marked ants shows that night ants and day ants do not have the same foraging time preference. As Fig. 3A–D shows, marked ants have a preference to forage in the same lighting conditions as during the marking day 0. The lighting condition is the defining factor influencing foraging behavior, rather than work shift. L2 and D1 ants prefer to forage in the same work shift as in day 0, however L1 and D2 ants prefer to forage during work shifts L2 and D1, respectively.

There is a striking feature about these results that must be addressed. Although literature^35,36^ and our data show that the main activity for the whole colony takes place in night phase, there were a considerable number of marked ants wandering both in L1 and L2 phases. There were also more ants marked in L1 and L2 than in D1 and D2. It seems contradictory, however, as Fig. 4 shows, there is a higher flow during night, but, fewer ants are engaged to foraging. Also, as Fig. 3 shows, although fewer ants were marked, night phases had higher reappearance of D1 and D2 ants. Thus, these collective pattern may have biased the number of marked ants, but, it only sustain our hypothesis about two different colony states, since it is a measure of differential ants engagement to foraging in different day phases.

Since the observed colonies revealed these two activity states that clearly correlated with phase, and that ants appearing in each state were also different in size, it is reasonable to assume that the colonies activity expresses a natural temporal division of labor in work shifts. This organization is particularly interesting for a system that works around the clock. Assuming the biological need to rest, ants that work nonstop would have a lower life span and a reduced task performance^43–45^. Therefore, division of labor in work shifts in leaf-cutting ants allows for a continuous colony activity that respects natural individual biological rhythms but assures efficiency of the colony.

Data shows a substantial difference in day-night ratio of foraging activity from laboratory to environmental conditions^36^. It may be possible that such variations could happen in nature as a response to seasonal variations. However, our experiments were carried in controlled conditions of photoperiod, temperature and humidity. This means that our results present an unbiased, unmasked expression of colony activity rhythm^27^. Hence the differences observed in natural and laboratory conditions.

Moreover, division of labor in work shifts of leaf-cutting ants is possible because there are individuals with particular associations between activity and lighting conditions. This was demonstrated in previous studies. Different *Camponotus* workers show a naturally expressed diurnal, nocturnal or even arrhythmic activity patterns, depending on the individual^37^. Also, they can adjust activity to address different feeding times and social contexts^46^. Similarly, *Diacamma sp.* nurses are able to change their activity pattern when paired to different brood types (i.e., eggs, larvae or pupae) ^47^. These data strongly suggest that activity rhythms in ants can be plastic and match either the internal state or environmental stimuli.

The mechanisms underlying division of labor in work shifts remain unknown, but some hypothesis can be extrapolated from other social insects. Honeybee nurses and foragers have a different relation between activity and environmental cycles: nurses are younger and work around the clock inside the beehives caring for the queen and juveniles, while foragers are older and diurnal, seeking and retrieving food outside the colony^48^. This pattern is closely related to worker’s physiology, for instance in nurses, clock genes are not expressed rhythmically^49,50^ and they have lower levels of juvenile hormone compared to foragers. On the other hand, foragers have a strong diurnal activity pattern, clock genes that are rhythmically expressed and higher levels of juvenile hormone^51^. It is worth pointing out that biological rhythms arise in foragers only when workers begin to face the cyclic environment. A similar physiological pattern could rule division of labor in leaf-cutting ants.

The workers patriline is another aspect to be considered in this discussion. As in honeybees, queens of leaf-cutting ants mate with several males before founding a colony (i.e. polyandry). This means that workers are offspring from the same mother but carry genes from different fathers. In honeybees this differential patriline influences in the workers’ time-foraging preference^52^.

Although the genetic aspects of behavior are promising, no gene was studied. The only difference detected between day (L1 and L2) and night ants (D1 and D2) is body size. Leaf-cutting ants have a high degree of polymorphism which implies differences in metabolism, physiology and behavior^18,39,40,53,54^. Day ants are bigger than night ants (Fig. 5). It is possible that this morphological difference reflects an adaptation to environmental conditions faced by workers during their foraging time. It is known that for insects, including ants, there is a relationship between body mass and thermal resistance: bigger bodies are more resistant to high temperatures and to dehydration than smaller bodies^55–57^. As *Atta sexdens* ants belong to a tropical environment and forage in long trails above the ground it is reasonable to hypothesize that bigger workers, whose thermal resistance is higher, were selected to forage during daytime while smaller workers, less tolerant to heat, were selected to forage during nighttime. Obviously, this hypothesis requires premises that natural selection acts on the individuals rather than on the colony itself, which is still an unsolved evolutionary challenge^58–61^.

Division of labor in work shifts allows a continuous exploitation of food sources. As Fig. 4 shows, despite small, there were always a number of ants returning to the nest carrying leaf fragments; this is compatible with field observations^35,36^. However, the colony’s foraging effort and efficiency were different at each time phase (3.4% during the dark phase; 5.4% during light phase). Even though more leaves were fetched during nighttime, the proportion of loaded ants returning to the nest was bigger during daytime (Fig. 5).

Moreover, previous studies show that foraging tasks are partitioned between two body size castes. Bigger ants frequently transport leaf fragments that were cut by other ants, while smaller ants cut and transport their own fragments^62^. As discussed above, bigger ants are present in trails more frequently during the light phase, which implies more transport. Meanwhile, during the night smaller ants could cut leaf fragments leaving them to be transported by other ants. Since night flow is higher, total leaf transport is expected to be higher as well. However, day flow is made by bigger ants, whose engagement in transport is proportionally bigger. Hence the pattern observed in Fig. 4; ants proportionally carrying more leaf during day than night, although the absolute number of leaf transported is bigger during night.

Division of labor in work shifts were described only in higher social organizations such as bees^43,49,52^, ants^37,47^ and humans^63^. There is a possibility that this pattern emerges only in social organizations due to an intrinsic need of nonstop activity (i.e. constant care of others). Therefore, it could be an adaptation to life in society.

Results presented here elucidate how leaf-cutting ants, whose foraging trails bear workers around the clock, are capable of dividing labor in work shifts. Such results were obtained observing workers foraging behavior within colonies in a controlled environment. It was possible to establish how workers with different activity patterns organize themselves during collective foraging. These data agree with previous results in leaf-cutting ants as well as other eusocial species, such as bees and other ant species. Our results contribute to a better understanding of how individuals within a colony can divide labor temporally opening possibilities for novel research in behavioral genetic and physiology of eusocial insects.
